# Estimation of genotoxicity, apoptosis and oxidative stress induction by TiO_2_ nanoparticles and acrylamide subacute oral coadministration in mice

**DOI:** 10.1038/s41598-022-23302-w

**Published:** 2022-11-04

**Authors:** Gehan Safwat, Amira A. Mohamed, Hanan R. H. Mohamed

**Affiliations:** 1 Zoology Department Faculty of Science, Cairo University, Giza, Egypt; 2 Faculty of Biotechnology, October University for Modern Sciences and Arts, Giza, Egypt

**Keywords:** Genetics, Molecular biology

## Abstract

Acrylamide is used in the industry and can be a by-product of high-temperature food processing which has toxic potential in various tissues, and titanium dioxide nanoparticles (TiO_2_NPs) are widely used in toothpaste, sweets, food perseveration, chewing gum and medicines. Consequently, humans are daily exposed to large amounts of acrylamide and TiO_2_NPs mainly through food intake. However, limited studies are available on the effect of simultaneously intake of acrylamide and TiO_2_NPs on the integrity of genomic DNA and the induction of apoptosis in brain tissues. Therefore, this study estimated the influence of acrylamide coadministration on TiO_2_NPs induced genomic instability and oxidative stress in the brain tissues of mice. To achieve this, mice were orally administrated acrylamide (3 mg/kg b.w) or/and TiO_2_NPs (5 mg/kg b.w) for two successive weeks (5 days per week). The comet assay results showed that concurrent oral administration of acrylamide and TiO_2_NPs strongly induced single- and double stranded DNA breaks, and that the level of reactive oxygen species (ROS) was also highly elevated within neural cells after simultaneous oral intake of acrylamide and TiO_2_NPs compared to those observed after administration of acrylamide or/TiO_2_NPs alone. Moreover, oral co-administration of acrylamide with TiO_2_NPs increased apoptotic DNA damage to neurons by upregulating the expression levels of P53, TNF-α, IL-6 and Presenillin-1 genes compared to groups administered TiO_2_NPs. Therefore, from these results, the present study concluded that coadministration of acrylamide renders TiO_2_NPs more genotoxic and motivates apoptotic DNA damage and oxidative stress induced by TiO_2_NPs in brain cells, and thus it is recommended to avoid concurrent oral acrylamide administration with TiO_2_NPs.

## Introduction

The effect of nanoparticles on human health has recently been the subject of much controversy. A total of 1814 nanotechnology-based items have been supplied to the global market, of which 117 are classified under the “Food and Beverage” category, according to the Nanotechnology Consumer Product Inventory^1^. Titanium dioxide nanoparticles (TiO_2_NPs) are one of the nanoparticles widely used in the preservation of foods and various consumer products such as sweets, chewing gum and cosmetics. TiO_2_NPs are also used as food additives in many food products such as skimmed milk, cheeses, pastries, ice-creams, and sauces^2–4^.

As a result of these intensive uses human daily ingestion of TiO_2_NPs is increased through oral intake of various foods and products containing TiO_2_NPs. Scientific interests in studying the bio-distribution and toxicities of TiO_2_NPs are growing. Several in vivo studies have demonstrated the distribution and entrance of TiO_2_NPs to the liver, kidney, lung, and even the brain by crossing the blood–brain barrier^2,5–7^**.** Upon entry, TiO_2_NPs attack and destroy the cell genome resulting in chromosomal abnormalities, DNA breakage and genes' mutation leading to apoptosis^3,6,8,9^.

Many toxic substances other than nanoparticles are also ingested by humans as a result of food processing and heat treatments that cause chemical transformation of food components. Examples for food-processing-induced toxins heterocyclic aromatic amines, advanced glycation end products, nitrosamines, and acrylamide. Acrylamide is a colorless and odorless by-product formed when foods containing high levels of carbohydrates and asparagine molecules are roasted at a certain temperature. A notable example of treatment-induced toxins is acrylamide^10,11^.

During high temperatures, above 120 °C, and low humidity, the amino acid asparagine reacts with reducing sugars such as glucose and fructose to form acrylamide. Acrylamide can be found in fried, baked and grilled dishes in the highest concentrations^12^. Clastogenicity and genotoxicity of acrylamide have been demonstrated in different experimental systems as manifested by the induction of chromosomal aberrations, DNA damage and mutations observed in mice and rats after administration of acrylamide^13–18^**.**

However, too limited studies are available on the effect of coadministration of acrylamide with TiO_2_NPs on the genomic DNA integrity and reactive oxygen species (ROS) generation in the brain tissues of mice. Thus, the current study was undertaken to estimate the impact of acrylamide concurrent administration with TiO_2_NPs on the genomic DNA integrity and oxidative stress induction in the brain tissue of mice. Alkaline comet and laddered DNA fragmentation assays was conducted to assess the integrity of genomic DNA. The levels of apoptotic genes expression and intracellular ROS were also studied using quantitative real time polymerase chain reaction (qRT-PCR) and 2,7 dichlorofluorescin diacetate dye, respectively.

## Materials and methods

### Chemicals

Acrylamide was purchased in the form of white powder from Sigma-Aldrich Chemical Company (St. Louis, MO, USA) and dissolved in deionized distilled water to prepare the administrated dose 3 mg/kg b.w that equivalent to human exposure dose^19^. Likewise, TiO_2_NPs was obtained in the form of white powder from Sigma-Aldrich Chemical Company (St. Louis, MO, USA) with particle size less than 100 nm and freshly suspended using ultra-sonication in deionized distilled water instantly before use to prepare the tested dose (5 mg/kg b.w) of TiO_2_NPs^20,21^ that equivalent to human exposure dose. The other used chemicals and reagents in the experiments were of analytical and molecular biology grade.

### Characterization of TiO_2_NPs

The purchased TiO_2_NPs with a purity of 99.5% and CAS number 13463-67-7 was well characterized using X-ray diffraction (XRD) to ensure the purity of the crystalline TiO_2_NPs and also the shape and average particle size of the suspended TiO_2_NPs were studied using transmission electron microscopy (TEM).

### Animals

Male Swiss Webster mice (20–25 g) were purchased from Animal House of the National Organization for Drug Control and Research (NODCAR), Giza, Egypt and were left for one week before treatment under a standard light–dark cycle to acclimatize to animal house conditions (12-h light cycle, 25 ± 2 °C temperature) with free access to standard rodent chow and water at the Department of Zoology, Faculty of Science, Cairo University.

### Ethical approval

The protocol and experimental design of this study have been reviewed and approved by the Institutional Animal Care and Use Committee (IACUC) at Cairo University with the accreditation number (CU/I/F/15/18). This study was reported according to ARRIVE guidelines and also Animal handling and experimentations were conducted in accordance with the Guidelines of the National Institutes of Health (NIH) regarding the care and use of animals for experimental procedures.

### Ethical approval for animals

This study was reported according to ARRIVE guidelines and the protocol and experimental design of this study have been reviewed and approved by the Institutional Animal Care and Use Committee (IACUC) at Cairo University with the accreditation number (CU/I/F/15/18).

## Experimental design

Twenty four male mice were randomly divided into four groups; each group contained six animals: the first group (Group I) was the negative control group in which the mice were orally given deionized distilled water. The second group (Group II) mice were given orally acrylamide at the exposure dose level of 3 mg/kg body weight^19^, while in the third group (Group III) mice were given orally 5 mg/kg body weight of TiO_2_NPs^20,21^ that equivalent to human exposure dose. Finally, acrylamide (3 mg/kg b.w) and TiO_2_NPs (5 mg/kg b.w) were orally coadministered to mice of Group IV simultaneously. Acrylamide or/and TiO_2_NPs were administered five times per week for two successive weeks and 24 h after the last administration the mice were sacrificed and dissected to obtain brain tissues, then stored at − 80 °C for further molecular studies.

### Alkaline comet assay to assess DNA breakages

The induction of DNA damage in the brain tissues of four groups was studied using alkaline Comet assay based on the protocol of Tice et al.^22^. About 100 mg of brain tissues were gently homogenized and 10 µl of clear suspensions containing about 10,000 cultured cells were mixed with 80 µl of 0.5% low melting point agarose. This mixture was then spread on a fully frosted slide which was fully pre-dipped in normal melting agarose (1%). After drying and hardening, the slides were placed in cold lysis buffer with freshly added 10% DMSO and 1% Triton X-100 for 24 h at 4ºC in darkness. Slides were then dipped in a freshly prepared alkaline buffer for 15 min and electrophoresed single-stranded DNA for 30 min at 25 V and 300 mA (0, 90 V/cm). The slides were neutralized in 0.4 M Trizma base (pH 7.5) to neutralize the alkali, fixed in absolute cold ethanol, left to air dry and finally, slides were stored at room temperature until they imaged and scored. For scoring slides were immediately stained with ethidium bromide (2 µg/ml) prior imaging, examined and photographed using epi-fluorescent microscope. The DNA migration for each sample was determined by simultaneous image capturing and scoring of 50 cells for each sample using TriTek Comet ScoreTM Freeware v1.5 scoring software that demonstrated some of the function of the Auto Comet(tm). Tail length, % DNA in tail and tail moment were used as indicators for the DNA in neural cells.

### Ladder DNA fragmentation assay

Induction of apoptotic DNA damage by acrylamide or/ and TiO_2_NPs was studied using laddered DNA fragmentation assay because DNA fragmentation is considered as one of the later steps in the apoptotic process. Using Sriram et al*.*,^23^ protocol: about 50 mg of brain tissues was homogenized in Tris EDTA (TE) lysis buffer containing 0.5% sodium dodecyl sulfate and 0.5 mg/ml RNase A, and then incubated at 37 °C for 1 h. Added Proteinase K (0.2 mg/ml), incubated the samples at 50 °C overnight, and DNA was then extracted and precipitated. The extracted DNA was electrophoresed in 1% agarose gel at 70 V and visualized using a UV trans-illuminator and photographed.

### Studying the intracellular ROS generation

Effect of acrylamide or/ and TiO_2_NPs administration on the level of intracellular ROS generation was studied using 2,7 dichlorofluorescin diacetate dye. This dye passively enters within cells and interacts with ROS forming the highly fluorescent compound dichlorofluorescein^24^. An equal volume of cells suspension and 2,7 dichlorofluorescin diacetate dye were gently mixed and incubated for 30 min in dark. Then this mixture was layered and spread on clean slide, and the cells were visualized and photographed using epi-fluorescence microscope at 20 × magnification.

### Measuring the expression levels of p53, IL-6, TNF-α and presenillin-1 genes

Quantitative Real Time Polymerase Chain Reaction (qRT-PCR) was conducted to estimate the effect of acrylamide or/and TiO_2_NPS administration on the expression levels of p53, Interleukin-6 (IL-6), Tumor necrosis factor-α (TNF-α), and Presenillin-1 genes in brain tissues of the control and treated groups. Whole cellular RNA was first extracted from brain tissue using Gene JET RNA Purification Kit and using the Nanodrop device the purity and concentration of the total extracted RNA were measured. Based the instructions of Revert Aid First Strand cDNA Synthesis Kit total extracted RNA was completely converted into complementary DNA (cDNA). For measuring the expression levels of P53, IL-6, TNF-alpha and Presenillin-1 genes a separate SYBR green based qRT-PCR was done for each sample using the previously designed primers shown in Table 1^25,26^**.** Duplicate of each group were done for each gene and the results of gene expression were normalized to β-actin as a housekeeping gene. Gene expression was quantified using the comparative Ct (DDCt) method and the fold values calculated using the formula: x = 2^(−DDCt)^.

**Table 1:** Sequences of the used primers in qRT-PCR.

Gene	Strand	Sequence
P53	Forward	5′ACC ATC GGA GCA GCC CTC AT 3′
	Reverse	5′TAC TCT CCT CCC CTC AAT AAG 3′
TNF-α	Forward	5′CCC GAG TGA CAA GCC TGT AG 3′
	Reverse	5′GAT GGC AGA GAG GAG GTT GAC 3′
IL-6	Forward	5′CAT GTT CTC TGG GAA ATC GTGG 3′
	Reverse	5′AAC GCA CTA GGT TTG CCG AGTA 3′
Presenillin-1	Forward	5′ AAA GGT CCA CTT CGA CTC CA 3′
	Reverse	5′ GGC ATT CCT GTG ACA AAC AA 3′
β-actin	Forward	5′TCA CCC ACA CTG TGC CCA TCT ACGA 3′
	Reverse	5′GGA TGC CAC AGG ATT CCA TAC CCA 3′

### Statistical analysis

Results of the current study are expressed as mean ± Standard Deviation (S.D) and were analyzed using the Statistical Package for the Social Sciences (SPSS) (version 20) at the significance level p < 0.05. One way analysis of variance (ANOVA) followed by Duncan's test was used to compare between the different treated groups and untreated control group.

## Results

### Characterization of TiO_2_NPs

**Figure 1:**
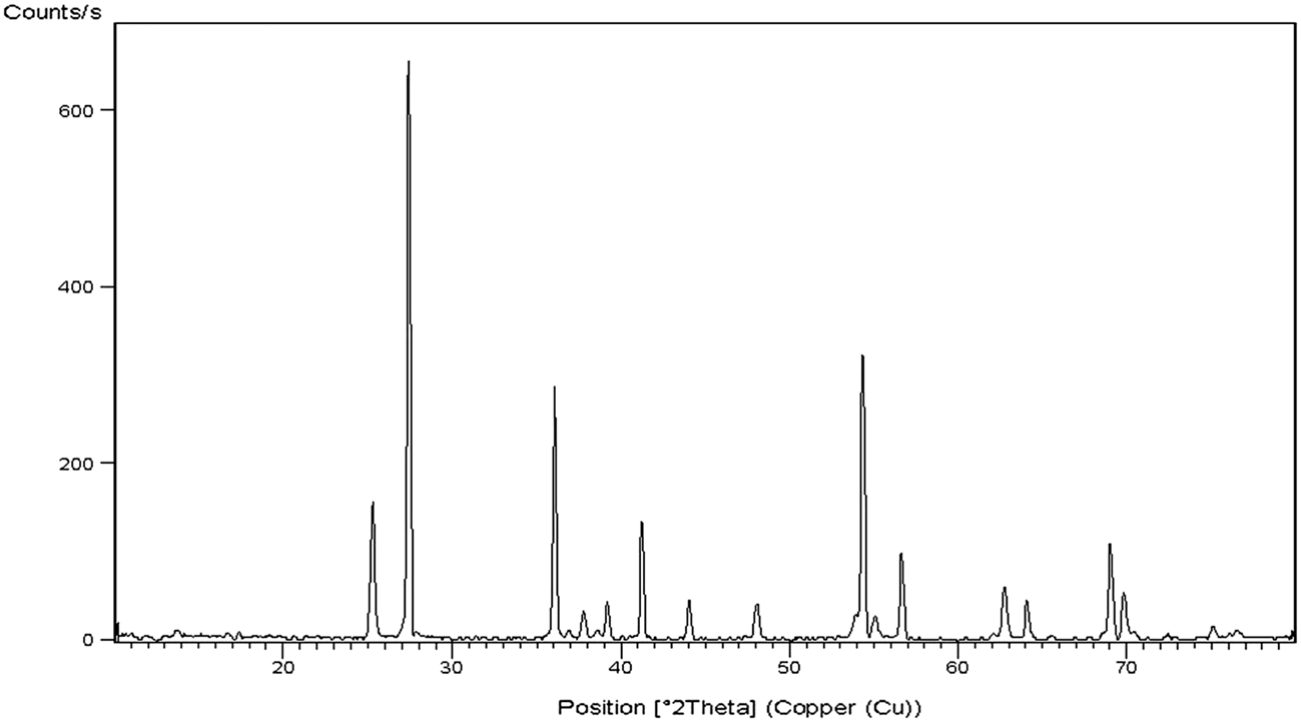
X-Ray Diffraction pattern (XRD) of TiO_2_NPs.

**Figure 2:**
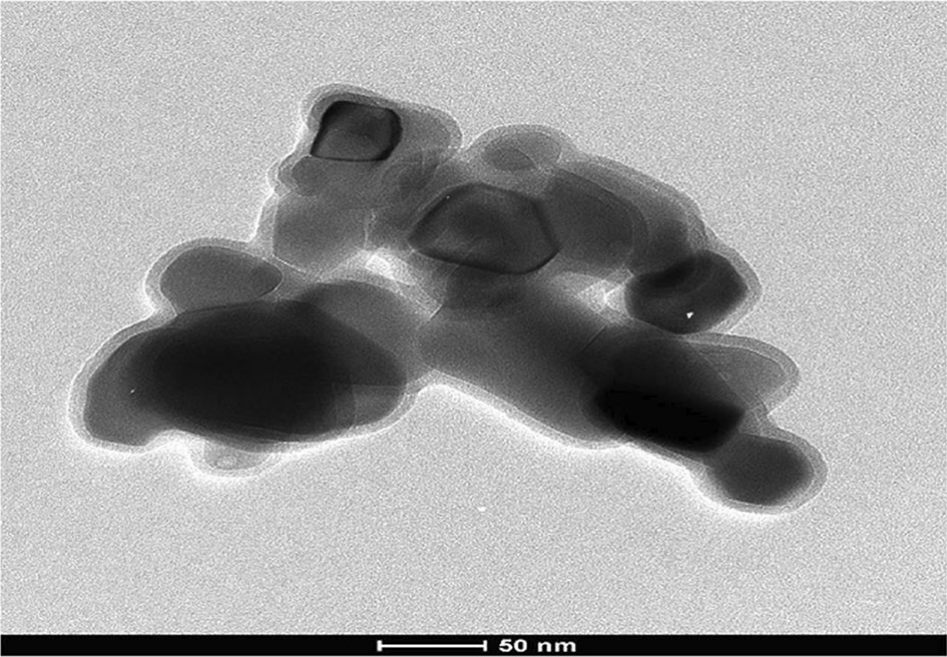
Transmission electron microscope imaging of TiO_2_NPs.

The obtained XRD pattern confirmed the purity of TiO_2_NPs as manifested from the appearance of the characteristic bands of TiO_2_NPs at angles of 25.2°, 27.8°, 36.1°, 41.2° and 54.7° shown in Fig. 1. Imaging with TEM revealed the polyhedral morphology of the suspended TiO_2_NPs with an average particle size of 60 nm (Fig. 2).

### Induction of DNA breaks

**Figure 3:**
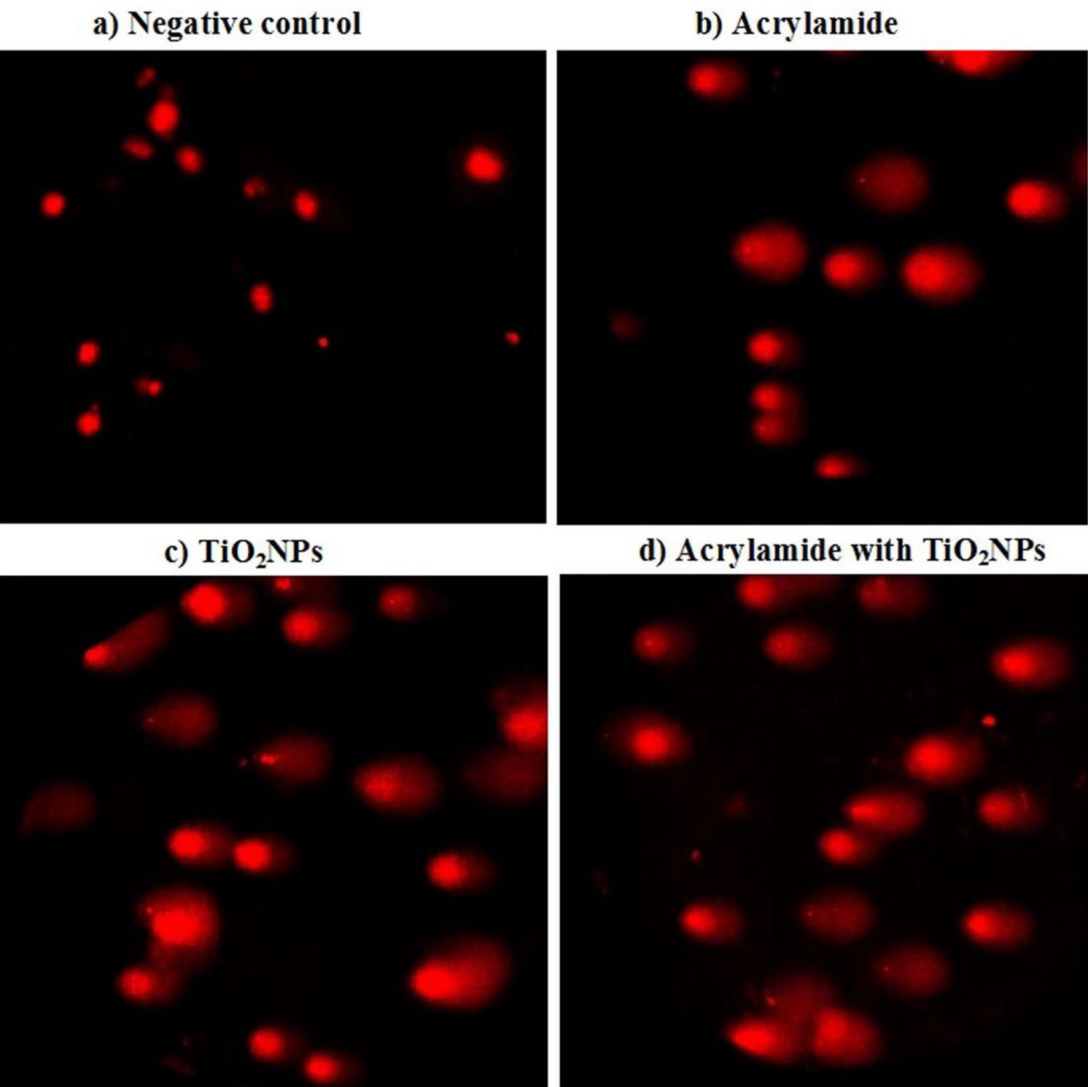
Representative photomicrograph for the observed comet nuclei in the brain tissues of (**a**) negative control group, (**b**) acrylamide administrated group, (**c**) TiO_2_NPs administrated group, and (**d**) group administered acrylamide with TiO_2_NPs.

As displayed in Table 2 oral administration of either acrylamide (3 mg/kg b.w) or TiO_2_NPs (5 mg/kg b.w) separately resulted in statistically significant increases in the tail length, %DNA in tail and tail moment compared to the negative control values. Furthermore, simultaneous co-administration of acrylamide with TiO_2_NPs caused a sudden DNA damage induction as determined by the high statistically significant increases in tail length, %DNA in tail and tail moment as compared to the negative control and TiO_2_NPs administered groups. The different degrees of DNA damage noticed in the brain tissues of both negative control and acrylamide or/ and TiO_2_NPs administered groups are shown in Fig. 3.

**Table 2:** Tail length (px), %DNA in tail and tail moment in the brain tissue of the negative control group and groups administered acrylamide or/and TiO_2_NPs.

Group	Treatment (dose mg/kg)	Tail length (px)	%DNA in tail	Tail moment
I	Negative control (deionized water)	3.93 ± 0.51^a^	10.18 ± 1.23^a^	0.42 ± 0.03^a^
II	Acrylamide (3 mg/kg)	7.12 ± 0.43^b^	17.73 ± 1.68^b^	1.27 ± 0.04^b^
III	TiO_2_-NPs (5 mg/kg)	9.09 ± 0.87^c^	26.45 ± 3.32^c^	2.42 ± 0.30^c^
IV	Acrylamide + TiO_2_-NPs	10.43 ± 1.70^c^	35.12 ± 2.90^d^	3.64 ± 0.31^d^
One Way Analysis of Variance		F = 23.05 P < 0.001	F = 57.74 P < 0.001	F = 126.39 P < 0.001

Results are expressed as mean ± SD. Results were analyzed using one-way analysis of variance followed by Duncan’s test to test the similarity between the control and three treated groups. Means with different letters indicates statistical significant difference between the compared groups in the same column.

### Ladder DNA fragmentation assay

**Figure 4:**
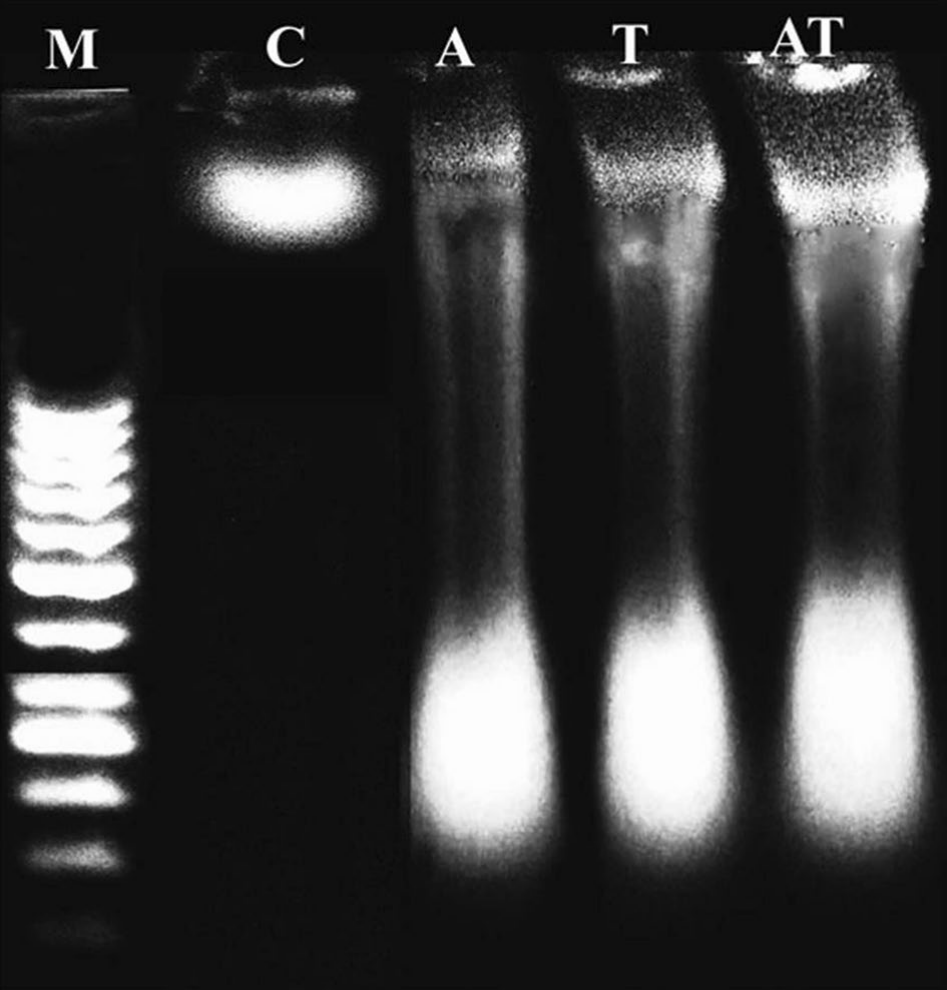
Electrophoresis pattern of the extracted genomic DNA from the negative control group (C) and groups administrated acrylamide (A), TiO_2_NPs (T), and acrylamide with TiO_2_NPs (AT) on an ethidium bromide stained agarose gel. M: Marker.

Screening the pattern of the electrophoresed genomic DNA on an ethidium bromide stained agarose gel demonstrated the induction of apoptotic DNA fragmentation by oral administration of acrylamide or/and TiO_2_NPs as manifested by the fragmentized and smeared appearance of the extracted genomic DNA from the brain tissues of mice given acrylamide or/and TiO_2_NPs compared with the intact appearance of genomic DNA extracted from rain tissue of control mice (Fig. 4).

### Level of intracellular ROS generation

**Figure 5:**
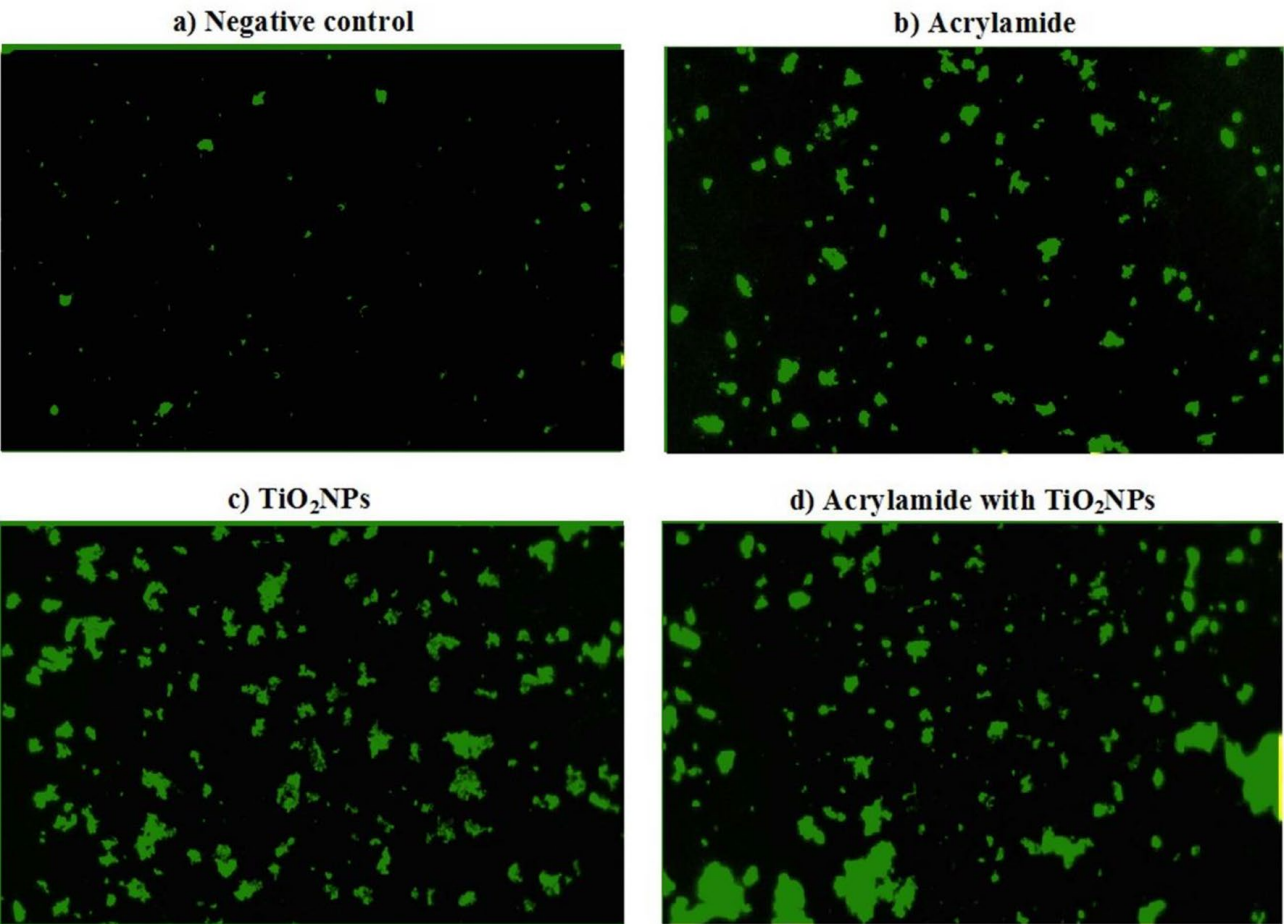
Level of intracellular ROS generation in the brain tissues of (**a**) negative control group, (**b**) acrylamide administered group, (**c**) TiO_2_NPs administered group and (**d**) group administered acrylamide with TiO_2_NPs.

As shown in Fig. 5, the high elevations induced in the ROS generation within neural cells by oral administration of either acrylamide or/and TiO_2_NPs were displayed by the observed high observable increases in the intensity of fluorescent light emitted from the stained neurons compared to that emitted from stained negative control cells. Moreover, concurrent administration of acrylamide with TiO_2_NPs induced marked increases in intracellular ROS generation as indicated by observed remarkable increases in the intensity of emitted fluorescent light emitted compared to that emitted from neural cells of mice given emitted TiO_2_NPs alone (Fig. 5).

### Genes' expression

As obvious in Table 3 oral administration of either acrylamide at the dose level 3 mg/kg or TiO_2_NPs at the dose level 5 mg/kg highly upregulated the expression levels of P53, TNF-α, IL-6 and Presenillin-1 genes compared to their expression levels in the negative control group. Furthermore, simultaneous co-administration of acrylamide with TiO_2_NPs significantly increased the expression level of P53, TNF-α, and IL-6 genes, and slightly increased the Presenillin-1 gene expression level compared to their expression levels in mice orally given TiO_2_NPs alone (Table 3).

**Table 3:** Expression level of p53, TNF-α, IL-6 and Presenillin-1 in the brain tissue of the negative control group and groups administered acrylamide or/and TiO_2_NPs.

Group	Treatment (dose mg/kg)	P53	TNF-α	IL-6	Presenillin-1
I	Negative control (deionized water)	1.00 ± 0.00^a^	1.00 ± 0.00^a^	1.00 ± 0.00^a^	1.00 ± 0.00^a^
II	Acrylamide (3 mg/kg)	4.23 ± 0.28^b^	2.71 ± 0.14^b^	3.35 ± 0.11^b^	2.32 ± 0.07^b^
III	TiO_2_-NPs (5 mg/kg)	2.12 ± 0.18^c^	2.06 ± 0.15^c^	2.36 ± 0.07^c^	1.94 ± 0.16^c^
IV	Acrylamide + TiO_2_-NPs	4.75 ± 0.24^d^	3.59 ± 0.17^d^	5.26 ± 0.23^d^	2.22 ± 0.10^b^
One Way Analysis of Variance		F = 214.55 P < 0.001	F = 215.37 P < 0.001	F = 541.18 P < 0.001	F = 105.51 p < 0.001

Results are expressed as mean ± SD. Results were analyzed using one-way analysis of variance followed by Duncan’s test to test the similarity between the control and three treated groups. Means with different letters indicates statistical significant difference between the compared groups in the same column.

## Discussion

Acrylamide is a common chemical found in molecular biology labs as well as a wide range of manufacturing and processing operations. For example, food cooked at high temperatures forms acrylamide thereby humans are exposed to acrylamide with widely used TiO_2_NPs in a variety of ways through their nutrition, work, leisure activities, and other daily routines and surroundings^27,28^. However, the impact of acrylamide coadministration with TiO_2_NPs on genomic integrity and apoptosis induction in brain tissue has not been well studied. Therefore, this study was conducted to estimate the influence of acrylamide coadministration on TiO_2_NPs induced genomic instability and apoptosis in mice brain tissue.

According to the results of the comet assay, the current study found that multiple oral administrations of either acrylamide (3 mg/kg b.w) or TiO_2_NPs (5 mg/kg b.w) exposure dose caused high DNA breakages as manifested by the remarkable statistically significant increase observed in comet parameters: tail length, %DNA in tail and tail moment compared to negative control values. These results confirmed the DNA damage induction by acrylamide^13,15–18^ or TiO_2_NPs^2,3,6,8,9^ reported in previous studies. Moreover, oral simultaneous coadministration of acrylamide with TiO_2_NPs augmented genomic DNA disruption and damage as indicated by the observed remarkable elevations in tail length, %DNA in tail and tail moment compared to their values in mice orally given TiO_2_NPs alone.

Acrylamide is completely soluble chemical and can cross blood brain barrier attacking brain cells inducing oxidative stress that weaken brain cells and make it more susceptible to TiO_2_NPs induced DNA damage [^29,30^]. Oxidative stress induction through extra ROS generation by acrylamide administration was manifested in this study from the high elevations in the intensity of florescent light emitted from brain cells stained with 2,7 dichlorofluorescin diacetate dye. Meanwhile, over generation of ROS induced by TiO_2_NPs alone within brain cells was augmented by coadministration of acrylamide with TiO_2_NPs.

Intracellular ROS are naturally generated during various metabolic processes. However, disturbance of balance between ROS generation and antioxidant defense system inducing oxidative stress^29–31^. Excessive ROS generation and DNA breakages trigger apoptosis of brain cells. Apoptosis induction by acrylamide and TiO_2_NPs administered separately or in combination was demonstrated by the fragmentized smeared appearance of genomic DNA on an ethidium bromide stained agarose gel in consistence with previous studies^3,32,33^. Ongoing with the results of ROS generation and DNA damage induction, apoptotic DNA damage induction was increased obviously in the genomic DNA of mice orally co-administrated acrylamide with TiO_2_NPs.

Moreover, over generation of ROS demonstrated within the brain cells of mice given acrylamide with TiO_2_NPs motivated inflammation through excessive release of pro-inflammatory cytokines^34,35^. Therefore, motivation of TiO_2_NPs induced inflammation in the brain tissue can be attributed to high increases in the expression levels of inflammatory IL-6 and TNF-α cytokines demonstrated after coadministration of acrylamide with TiO_2_NPs compared to their expression in mice given acrylamide or TiO_2_NPs separately.

In response to a variety of cellular stressors such as oxidative stress, hypoxia, DNA damage, ribonucleotide depletion, and oncogene activation, the tumor suppressor p53 gene is activated and overexpressed motivating apoptosis^36–39^. Consequently, the augmentation of TiO_2_NPs induced apoptosis noticed in this study may be due to the high increases demonstrated in the expression level of p53 after coadministration of acrylamide with TiO_2_NPs compared to its expression in mice given TiO_2_NPs or acrylamide separately.

The classical proinflammatory cytokine interleukin-6 (IL-6) performs crucial functions in the development, differentiation, regeneration, and degeneration of neurons in the nervous system. However, as a molecule, IL-6 has the potential to be both useful and harmful. IL-6 is capable of exerting activities that are diametrically opposed to one another, such as promoting neuronal survival after injury or contributing to neuronal degeneration and cell death in conditions such as Alzheimer's disease. Immune cells, such as macrophages, glial cells, and neurons, are some of the primary places where IL-6 is synthesized^40,41^. Several neural diseases such as Alzheimer's, Parkinson's and Huntington's diseases, brain cancer and ischemic stroke have all been linked to elevated IL-6 expression and secretion. Previous studies have shown that IL-6 is involved in the development of several disorders^42^. Similarly, level of TNF-α gene expression has been shown to elevate in Alzheimer's, and Parkinson's brain tissue, plasma, and cerebrospinal fluid^43^.

Presenilin1 is encoded by the gene Presenillin-1 and is responsible for the cleavage of the amyloid protein precursor that causes Alzheimer's disease. Presenilin-1 gene has been shown to be overexpressed in Alzheimer's disease^44^. Our finding of high increases in the expression level of presenillin-1 gene after coadministration of acrylamide with TiO_2_NPs may be due to the upregulation of IL-6 and TNF-α inflammatory mediators compared to its expression in the brain tissue of mice given TiO_2_NPs only. These results are consistent with previous studies^45,46^ that showed that overexpression of inflammatory mediators increase the expression level of β-amyloid precursor protein and Presenillin-1 genes that cause aggregation and accumulation of β-amyloid peptides in the brain tissues.

## Conclusion

Based on the afore discussed results, we concluded that oral simultaneous coadministration of acrylamide motivated TiO_2_NPs induced genomic instability through increasing intracellular ROS generation and the expression level of inflammatory cytokines and tumor suppressor p53 gene. More in vivo and in vitro studies using fluorescent nanoparticles, electron microscope and mass spectrometry are thus recommended to shed more light on the effect of acrylamide coadministration on TiO_2_NPs induced toxicity in the brain tissue.

## Data Availability

The datasets used and/or analyzed during the current study are available from the corresponding author on reasonable request.
